# Quality assessment of nine paracetamol 500 mg tablet brands marketed in Saudi Arabia

**DOI:** 10.1186/s13104-021-05672-y

**Published:** 2021-06-30

**Authors:** Reem AlSwayeh, Syed N. Alvi, Muhammad M. Hammami

**Affiliations:** 1 Clinical Studies and Empirical Ethics Department, King Faisal Specialist Hospital and Research Centre, P O Box # 3354 (MBC 03), Riyadh, 11211 Saudi Arabia; 2 Alfaisal University College of Medicine, Riyadh, Saudi Arabia

**Keywords:** Paracetamol, Pharmaceutical quality, Saudi market, Dissolution profile, Generic brands, Reference brands, Combinational drugs

## Abstract

**Objective:**

To evaluate in-vitro quality of paracetamol 500 mg tablet brands marketed in Saudi Arabia.

**Results:**

Two reference (R1 and R2) and seven generic (G1-G7) brands were commercially available. Four brands were single-drug, containing paracetamol only (R1, G1-G3) and five contained additional active ingredients (R2, G4-G7). All brands were immediate-release. Weight variation (n = 20, range as percent difference from mean), active substance content (n = 20, mean (SD) as percent difference from label), breaking force (n = 10, mean (SD)), and friability (n = 20, as percent weight loss) ranged from 97 to 102%, 96.1% (2.9%) to 99.8% (1.1%), 9.9 (0.4) to 21.0 (0.9) kg, and 0.017% to 0.809%, respectively. Disintegration (water medium) time (n = 6, minute: second) ranged from 02:35–03:09 to 12:49–13:10. Dissolution (phosphate buffer, pH 5.8) profile showed a mean release at 30 min of 87% to 97% of label content, with seven brands passing stage-1 (≥ 85% for each of 6 test units) and two passing stage-2 (mean of 12 test units ≥ 85%) criteria. Despite statistically significant differences between R1 and R2 and some of their corresponding generic brands in active substance content, breaking force, and amount dissolved at 30 min, all nine brands met the pre-specified quality standards.

**Supplementary Information:**

The online version contains supplementary material available at 10.1186/s13104-021-05672-y.

## Introduction

The World Health Organization, among others, has continuously advocated the use of generic drug products because they expand healthcare accessibility [1]. However, lay people [2] and occasionally healthcare workers [3] not infrequently view local drug products as inferior to imported ones, questioning their quality and interchangeability.

Most countries, including Saudi Arabia, require that generic drug products pass standard in-vivo bioequivalence testing before marketing [4]. Although this should be assuring, ongoing evaluation of marketed products is critical to maintain trust. Performing such evaluation in-vitro is gaining regulatory support and has the advantages of saving money and time and of not requiring involvement of human research subjects [5–8].

Paracetamol (acetaminophen) is a cheap, widely used analgesic and antipyretic drug that is available over-the-counter [9]. It is commercially available for oral intake as immediate-release tablets of various strengths, alone or in combination with other active ingredients [10].

We previously reported the pharmaceutical quality of various diclofenac tablet formulations available on the Saudi market [11, 12]. The aim of this study was to evaluate in-vitro quality of paracetamol 500 mg tablet brands marketed in Saudi Arabia.

## Main text

### Drugs and chemicals

We assessed all single-drug and combinational brands of paracetamol 500 mg immediate-release tablet that were commercially available in Riyadh, Saudi Arabia at the time of the study (September to December 2020). We were able to locate two reference (R1 and R2) brands and seven generic (G1-G7) brands. R1 and G1-G3 contained only paracetamol, while R2 and G4-G7 contained other active ingredients (caffeine, codeine, pseudoephedrine, or diphenhydramine). Label information of the studied brands is presented in Additional file 1: Table S1: Label information.

We purchased paracetamol (acetaminophen) reference standard from Sigma-Aldrich CO., Louis, MO., USA; HPLC grade methanol and acetonitrile from Fisher Scientific, Fair Lawn, NJ, USA; and sodium phosphate monobasic and sodium phosphate dibasic from Fisher Chemical, Fair Lawn, New Jersey, USA. HPLC grade water was prepared by reverse osmosis and was further purified by passing through a Milli-Q System (Millipore, Bedford, MA, USA).

### Instruments

High performance liquid chromatography (HPLC)-dissolution system consisted of Waters 2690D Separation Module, Hanson Research SR8-Plus, United States Pharmacopoeia (USP) dissolution apparatus II (paddle), and Waters 996 photodiode array detector (Waters Associates Inc., Milford, MA. USA). Mettler AT20 sensitive balance was purchased from Mettler Toledo (Greifensee, Switzerland). Model SSE-731 Microprocessor Disintegration Test Apparatus, Model SSE-710 Microprocessor Friability Apparatus, and Model SSE-DIGIT AB-SPV Digital Tablet Hardness Tester were purchased from Sunshine Scientific Equipment, Delhi, India.

### Sample preparation & HPLC assay

We prepared paracetamol stock solution in water and diluted it to produce standard curve samples of 40, 80, 160, 240, 320, 480, 560, and 700 µg/ml, and quality control samples of 40, 120, 350, and 630 µg/ml. A previously reported HPLC assay [13] was used to determine active substance content (ASC) and dissolution profiles. The assay uses Symmetry Shield RP18, 5 µm cartridge and a mobile phase composed of water, methanol, and acetonitrile (80:10:10, v: v: v), delivered at a flow rate of 0.9 ml/minute. The analysis was performed under isocratic condition (column and sample compartment temperature 40 °C and 8 °C, respectively). The photodiode array detector was set at 245 nm. There was no interference from tablet’s excipients. We used a standard curve with eight non-zero points (40, 80, 160, 240, 320, 480, 560, 700 µg/ml) and three sets of four quality control samples (40, 120, 350, 630 µg/ml) in each run. Mean (SD) accuracy and coefficient of variation (n = 9) were 103.6% (2.4%) and 5.3% (2.9%), 99.1% (5.0%) and 2.9% (1.8%), 99.6% (3.9%) and 3.5% (2.0%), and 102.3% (2.7%) and 3.0% (2.7%) for the four quality control samples, respectively.

### Quality control tests & calculations

For weight variation, friability, and ASC tests 20 randomly-selected units of each brand were examined. For the weight variation test, we calculated mean (SD) and percent deviation of individual unit’s weight from mean weight of the brand. For the friability test, units were weighted, placed in a friabilator operated at 25 revolutions/minute for 4 min, then weighted again after de-dusting. We determined friability as percent weight loss. For the ASC test, the units were individually crushed, dissolved in 100 ml water, filtered with a syringe using 0.2 µm filter, and diluted in water, and 20 µl were injected into the HPLC system. We calculated mean (SD) content and percent deviation of individual units from label.

For tablet breaking force test, 10 randomly-selected units of each brand were examined and mean (SD) pressure required to break each unit was determined.

Six randomly-selected units of each brand underwent disintegration testing. We placed the basket rack in a 1000 ml vessel containing 900 ml water (37 ± 2 °C). The basket rack moved 5–6 cm up and down (31 cycles/minute) with the test unit remaining 1.5 cm below liquid surface and 2.5 cm above beaker bottom. We determined range of disintegration time (time to no particle on the basket).

Eight randomly-selected units of each brand initially underwent dissolution testing. If one or more units failed, additional 4 units were examined. 900 ± 1% ml of phosphate buffer (pH 5.8) was used as dissolution medium. It was composed of 0.05 M sodium phosphate dibasic and 0.05 M sodium phosphate monobasic (50: 50, v: v). Temperature was maintained at 37 ± 0.5 °C using constant bath temperature. Stirring rate was 50 ± 1 rounds per minute (rpm) for 60 min and then the test ended with a stirring rate of 150 rpm for 15 min (“infinity”). One ml sample was withdrawn midway between dissolution medium surface and rotating blade top, ≥ 1 cm away from vessel wall, and was immediately replaced with fresh medium. The samples were withdrawn at 5, 10, 15, 20, 30, 40, 60, and 75 min. We kept the vessels covered, verified mixture temperature, and observed unit’s behavior throughout the test. We injected 20 µl of the one ml samples into the HPLC system. We determined mean (SD) amount released and percent of label ASC released, at each time point.

We compared ASC, breaking force, and amount dissolved at 30 min of R1 to G1, G2, and G3 (each contains paracetamol only) and of R2 to G4, G5, G6, and G7 (each contains paracetamol plus other active substances) using unpaired t test. Two tailed p value < 0.05 was considered significant.

### Results

Table 1 summarizes the main results of the study. Mean weight of the nine brands ranged from 553.2 (3.3) to 838.1 (10.5) mg. Weight range was within 97–102% of mean weight for each brands. Mean (SD) ASC ranged from 96.1% (2.9%) to 99.8.2% (1.1%). Both parameters were within the acceptable limits (≤ ± 5% and 90–110%, respectively) for each brand. However, R1 ASC was significantly lower than G1 ASC (p = 0.0002) and G2 ASC (p = 0.03), and R2 ASC was significantly higher than G4 ASC (p = 0.0001).

**Table 1:** In-vitro quality of two reference and seven generic paracetamol 500 mg tablet brands available on the Saudi market

Code	Weight n = 20		Active substance content^b^ n = 20		Breaking force n = 10	Friability n = 20	Disintegration^d^ n = 6	Dissolution^e^ n = 8
	Mean (SD), mg	Range^a^, % from mean	Mean (SD), mg	Mean (SD), % of label	Mean (SD), kg	% loss	Range minute: second	Mean (range) release at 30 min, % of label
R1	599.6 (4.1)	99–101	480.4 (14.6)	96.1 (2.9)	15.4 (0.7)	0.017	04:21–04:58	97 (96–98)
G1	569.4 (4.3)	98–102	497.8 (12.3)	99.6 (2.5)	16.3 (1.5)	0.625	02:35–03:09	91 (88–93)
G2	560.6 (4.3)	99–101	489.9 (12.8)	98.0 (2.6)	18.8 (1.2)	0.809	04:23–04:56	95 (92–97)
G3	553.2 (3.3)	99–101	485.9 (4.9)	97.2 (1.0)	13.1 (0.9)	0.378	04:42–05:35	97 (94–100)
R2	688.4 (5.8)	99–101	498.9 (5.7)	99.8 (1.1)	14.9 (0.9)	0.366	05:16–07:51	95 (94–97)
G4	744.1 (5.7)	99–101	488.6 (6.0)	97.7 (1.2)	9.9 (0.4)	0.717	10.39–10.59	87 (80–96)^f^
G5	560.6 (4.3)	99–101	497.5 (4.2)	99.5 (0.8)	18.3 (1.7)	0.351	8:43–9:30	96 (91–99)
G6	797.3 (4.5)	99–101	498.0 (5.4)	99.6 (1.1)	15.3 (1.5)	0.526	9:02–10:13	96 (94–98)
G7	838.1 (10.5)	97–102	498.1 (4.6)	99.6 (0.9)	21.0 (0.9)	0.167	12:49–13:10	96 (82–100)^f^

^a^Acceptable variation limits, ≤  ± 5% (for tablets ≥ 250 mg). ^b^Acceptable limits, mean content 90–110% of label. ^c^Acceptable limit, ≤ 1%. ^d^Water medium, acceptable limit, ≤ 15 min. ^e^Phosphate buffer (pH 5.8) medium, acceptable limit, ≥ 80 + 5% of label. ^f^Mean (range) of 12 rather than 8 units

Mean (SD) breaking force ranged from 9.9 (0.4) to 21.0 (0.9) kg. R1 breaking force was significantly lower than G2 breaking force (p = 0.0001) and significantly higher than G3 breaking force (p = 0.0001). On the other hand, R2 breaking force was significantly higher than G4 breaking force (p = 0.0001) and significantly lower than G5 braking force (p = 0.0001) and G7 breaking force (p = 0.0001). Weight loss due to friability ranged from 0.017% to 0.809%, which is within the acceptable limit of ≤ 1%.

Disintegration time was variable. It ranged from 02:35 to 03:09 min: second for G1 to 12:49 to 13:10 min: second for G7. Nevertheless, all integration times were within the acceptable limit of ≤ 15 min.

**Fig. 1:**
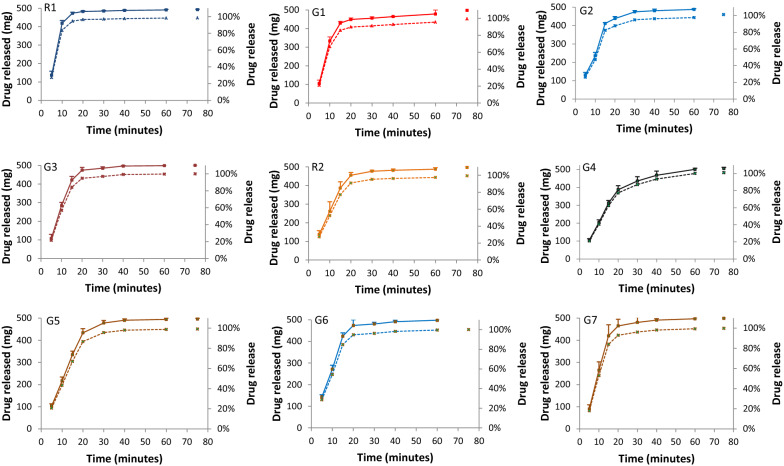
Dissolution profiles of two references and seven generic paracetamol 500 mg tablet brands available on the Saudi market. Mean (SD) amount of drug released at the specified times are shown on the left axis (continuous line) and percent of label amount released on the right axis (interrupted line). United States Pharmacopoeia (USP) dissolution apparatus type II (paddle apparatus) was used with a stirring rate of 50 ± 1 rpm (except for “infinity” time) and a temperature of 37 ± 0.5 ºC. Brands’ label details are available in Additional file 1: Table S1. Label information. R1 and R2, reference brands; G1 to G7, generic brands. Dissolution medium was phosphate buffer (pH 5.8). Time 75 min indicates amount released with a stirring rate of 150 rpm for 15 min (“infinity”)

Dissolution profiles of R1, R2 and G1-G7 are shown in Fig. 1. Each of the 8 units of all brands except G4 and G7 released ≥ 85% (Q (80%) + 5%) of label ASC within 30 min and thus met the acceptance criteria of stage-1 (≥ 85% release from each of 6 test units). [14] However, because 4 and 1 of the 8 units of G4 and G7, respectively, released < 85% of label ASC by 30 min (80–84% and 82%, respectively), an additional 4 units were tested for each brand. Mean percent release for 12 units of G4 and G7 was 87% and 96%, respectively, meeting the acceptance criteria of stage-2 (mean release of 12 test units ≥ 85%) [14]. During dissolution, we did not observe any artifact such as coning, gumming, capping, sticking, etc. for any of the brands. Interestingly, amount dissolved at 30 min was significantly higher for R1 compared to G1 (mean (95% confidence interval), 29.2 (23.2 to 35.2) mg, p = 0.0001) and G2 (mean difference 10.1 (4.0 to 16.2 mg, p = 0.003). Amount dissolved at 30 min was also significantly higher for R2 compared to G4 (mean difference 40.8 (22.7 to 58.9) mg, p = 0.0002).

### Discussion

We assessed the pharmaceutical quality of 2 reference and seven generic paracetamol 500 mg immediate-release tablet brands that were commercially available on the Saudi market at the time of the study. All brands passed in-vitro quality testing according to USP [15]. Namely, weight variation of ≤  ± 5% from mean weight (since tablet weight was ≥ 250 mg); mean ASC between 90–110% of label; ≤ 1% weight loss due to friability; complete disintegration in water within 15 min; and release of ≥ 85% within 30 min in phosphate buffer (pH 5.8).

The reported quality of paracetamol tablets on other markets is variable [16–18]. As examples, Zaid et al. evaluated the quality of 10 paracetamol products on the Palestinian market and concluded that generic products are often in-vitro comparable to the innovator product [16]. Similar conclusion was reached by Ukwueze et al. after studying six brands of paracetamol generic formulations marketed in Nigeria [17]. However, in a study from Malawi, out of 11 paracetamol brands, one failed ASC testing, and three failed friability testing [18].

The current results together with the results of in-vitro quality studies of drug products containing other active ingredients, [11, 12] in-vivo pre-marketing studies [19–26], and in-vivo post-marketing study [27] provide assurance of the quality of generic drug products commercially available in Saudi Arabia.

## Study strengths

The study assessed all commercially available brands, included two reference brands, examined multiple-point dissolution curves, and used a validated HPLC assay.

## Study limitations

Our results do not apply to other paracetamol 500 mg tablet brands that are listed on the Saudi formulary but were not commercially available at the time of the study. Further, our results do not apply to paracetamol formulations of other strength or form. Furthermore, the specific variations in manufacturing procedures that led to the observed differences among the different brands were not addressed in this study.

## Supplementary Information

**Additional file 1: Table S1.** Label information of two reference and seven generic paracetamol 500 mg tablet brands available on the Saudi market.

## Data Availability

Additional data are available in Additional file 1: Table S1. Label information. The datasets used and/or analyzed during the current study are available from the corresponding author on reasonable request.

## Abbreviations

ASC: Active substance content
G: Generic brand
HPLC: High performance liquid chromatography
R: Reference brand
rpm: Rounds per minute
SD: Standard deviation
USP: United States Pharmacopoeia
