# Supplementary material for: Quality assessment of nine paracetamol 500 mg tablet brands marketed in Saudi Arabia
Source: BMC Res Notes. 2021 Jun 30;14:254. doi: 10.1186/s13104-021-05672-y (PMC8247153; doi:10.1186/s13104-021-05672-y)
Supplement: Supplementary file 1 — Additional file 1: Table S1. Label information of two reference and seven generic paracetamol 500 mg tablet brands available on the Saudi market. [file 13104_2021_5672_MOESM1_ESM.docx]

**Additional file 1: Table S 1:** Label information of two reference and seven generic paracetamol 500 mg tablet brands available on the Saudi market

| **Code** | **Manufacturer** | **Manufacture date** | **Expiration date** | **Batch /lot number** | **Date Assessed** | **Active substance** | **Trade name** | **Form** | **Dose**  **(mg)** |
| --- | --- | --- | --- | --- | --- | --- | --- | --- | --- |
| R1 | GlaxoSmithKline, Dungarvan Ltd., Ireland | 2/2020 | 1/2024 | VH3P | 9/2020 | Paracetamol | Panadol | Tablet | 500 |
| G1 | SPIMACO, Al-Qassim Pharmaceutical Plant, KSA | 1/2020 | 1/2024 | 121115 | 10/2020 | Paracetamol | Fevadol | Tablet | 500 |
| G2 | National Pharmaceutical Industries Co. (SAOG), Oman | 2/2020 | 2/2023 | 0220001 | 10/2020 | Paracetamol | Omol | Tablet | 500 |
| G3 | Kuwait Saudi Pharmaceutical Industries Co. | 2/2020 | 2/2024 | KT89 | 10/2020 | Paracetamol | Panadrex | Tablet | 500 |
| R2 | GlaxoSmithKline, Dungarvan Ltd., Ireland | 10/2019 | 9/2023 | H59V | 10/2020 | Paracetamol/  caffeine | Panadol Extra | Tablet | 500/65 |
| G4 | SPIMACO, Al-Qassim Pharmaceutical Plant, KSA | 6/2020 | 6/2024 | 124694 | 11/2020 | Paracetamol/  Caffeine | Fevadol Extra | Tablet | 500/65 |
| G5 | National Pharmaceutical Industries Co. (SAOG), Oman | 3/2020 | 3/2022 | 3520008 | 11/2020 | Paracetamol/  Caffeine | Omol Extra | Tablet | 500/65 |
| G6 | SPIMACO, Al-Qassim Pharmaceutical Plant, KSA | 06 2020 | 06 2025 | 124600 | 11/2020 | Paracetamol/  Caffeine/Codeine | Fevadol Plus | Tablet | 500/30/8 |
| G7 | SPIMACO, Al-Qassim Pharmaceutical Plant, KSA | 03 2020 | 03 2023 | 122520 | 12/2020 | Paracetamol/  Pseudoephedrine/ Diphenhydramine | Flutab | Tablet | 500/30/25 |
